# The Impact of Neonatal Methamphetamine on Spatial Learning and Memory in Adult Female Rats

**DOI:** 10.3389/fnbeh.2021.629585

**Published:** 2021-02-18

**Authors:** Ivana Petrikova-Hrebickova, Maria Sevcikova, Romana Šlamberová

**Affiliations:** Department of Physiology, Third Faculty of Medicine, Charles University, Prague, Czechia

**Keywords:** methamphetamine, neonatal exposure, Morris Water Maze (MWM), strategies, Wistar rat

## Abstract

The present study was aimed at evaluating cognitive changes following neonatal methamphetamine exposure in combination with repeated treatment in adulthood of female Wistar rats. Pregnant dams and their pups were used in this study. One half of the offspring were treated indirectly *via* the breast milk of injected mothers, and the other half of pups were treated directly by methamphetamine injection. In the group with indirect exposure, mothers received methamphetamine (5 mg/ml/kg) or saline (1 ml/kg) between postnatal days (PD) 1–11. In the group with direct exposure, none of the mothers were treated. Instead, progeny were either: (1) treated with injected methamphetamine (5 mg/ml/kg); or (2) served as controls and received sham injections (no saline, just a needle stick) on PD 1–11. Learning ability and memory consolidation were tested on PD 70–90 in the Morris Water Maze (MWM) using three tests: Place Navigation Test, Probe Test, and Memory Recall Test. Adult female progeny were injected daily, after completion of the last trial of MWM tests, with saline or methamphetamine (1 mg/ml/kg). The effects of indirect/direct neonatal methamphetamine exposure combined with acute adult methamphetamine treatment on cognitive functions in female rats were compared. Statistical analyses showed that neonatal drug exposure worsened spatial learning and the ability to remember the position of a hidden platform. The study also demonstrated that direct methamphetamine exposure has a more significant impact on learning and memory than indirect exposure. The acute dose of the drug did not produce any changes in cognitive ability. Analyses of search strategies (thigmotaxis, scanning) used by females during the Place Navigation Test and Memory Recall Test confirmed all these results. Results from the present study suggested extensive deficits in learning skills and memory of female rats that may be linked to the negative impact of neonatal methamphetamine exposure.

## Highlights

- Neonatal exposure to MA (PD 1–11) impaired water maze performance of female rats in adulthood relative to saline exposed neonates (drug effect).
- Directly injecting the females with MA during PD 1–11 had a greater effect on adult performance during the acquisition and recall phases than indirect exposure through breast milk. These deficits were associated with an increase in thigmotaxic behaviors.
- Daily MA injections to adult female rats did not affect performance in the water maze.

## Introduction

Molecular mechanisms underlying memory formation include many specific signaling pathways involving neurotransmitter release, calcium influx, and second messenger activation, transcription of genes, *de novo* protein synthesis, and histone modification of gene expression (Keiser and Tronson, 2016). Each of these transduction pathways may be disrupted with methamphetamine (MA), a psychostimulant drug that primarily blocks dopaminergic and serotonergic systems (Sulzer et al., 2005). MA exposure leads to depletion of monoamines and their metabolites, which results in an irreversible decrease in the number of transporters, nerve terminals, and neuron cell bodies, not only in adulthood but also in the developing brain (Homer et al., 2008). MA exposure administered during brain development leads to decreased connectivity within DA-rich areas within the hippocampus, the amygdala, some parts of the cerebellum, and medial prefrontal cortex, which are structures important for learning and memory (Rice and Barone, 2000; Roussotte et al., 2011, 2012). Most hippocampal pyramidal cells are generated prenatally in the rat from gestation day (GD) 14 to 21–22, with about 15% of granulate cells of the dentate gyrus forming to postnatal days (PD) 19–21 (Rice and Barone, 2000). Hippocampal neurogenesis peaks in humans around the 8th gestational week, with up to 80% of dentate gyrus granule cells forming just before delivery (40th gestational week) (Rakic and Nowakowski, 1981; Clancy et al., 2007). The developmental differences between rats and humans were taken into account, birth in humans corresponds to the PD 10–12 in rats (Clancy et al., 2007). Studies from our laboratory have demonstrated that prenatal/neonatal and acute MA-induced impairments of brain regions involved in declarative memory function are time-dependent. While prenatal MA exposure (5 mg/ml/kg) during the entire gestation does not affect cognition in adult male and female rats (Schutová et al., 2009; Hrebíčková et al., 2014; Macúchová et al., 2017), administration of MA (5 mg/ml/kg) to mothers throughout the lactation period impairs cognition of their adult male offspring (Hrubá et al., 2010). The finding that the early postnatal period is more sensitive to MA-induced changes was recently confirmed by numerous studies (e.g., Williams et al., 2003a; Shansky and Woolley, 2016). A study by Hrebíčková et al. (2016) demonstrated that MA in dose 5 mg/ml/kg given during PD 1–11 affects cognitive functions of male rats in adulthood. The study compared the effect of MA exposure during different stages of rat brain development (i.e., the first half of gestation, the second half of gestation, and early neonatal stage). Our results suggested that the most serious impact of MA exposure was on hippocampal-dependent spatial learning [Morris Water Maze (MWM)] was associated with neonatal administration.

On the other hand, there are also studies from our laboratory that showed changes in cognition function not only after prenatal and neonatal MA exposure but also that learning and memory may be modified by repeated MA treatment exposed in adulthood, too. This sensitizing effect of MA, which seems to be caused by increased dopamine (DA) levels in structures of the mesolimbic system of the brain (Bubeníková-Valešová et al., 2009; Schutová et al., 2009, 2013; Fujáková-Lipski et al., 2017) suggests that animals with MA treatment in adulthood memorized the location of the platform most accurately. Other studies investigating the effect of chronic MA applications, in which acute MA in lower doses (0.1–0.4 mg/kg) was shown to produce improvements in cognitive processing when given to drug-naïve subjects (Kornetsky et al., 1959; Grilly and Loveland, 2001). In experimental animals, acute treatment with MA in a dose of 3 mg/kg disorders spatial and non-spatial memory was accompanied by loss of dopaminergic and serotonergic nerve terminals in the brain (Grilly and Loveland, 2001; Schroder et al., 2003). Based on these facts, the aim of this study is to examine the potential interaction of neonatal MA exposure during PD 1–11 and an acute dose of the same drug (1 mg/kg) in adulthood.

Our previous and recent studies demonstrated that there is a difference between the effect of MA administered to pups directly by injection or indirectly *via* maternal breast milk (injection to mothers; Hrebíčková et al., 2016, 2017; Ševčíková et al., 2017). Cognition in adult male rats has also been shown to be affected differently by direct and indirect drug administration (Hrebíčková et al., 2016). However, there is little research reporting on the transfer of MA through breast milk (Bartu et al., 2009). A study by Rambousek et al. (2014) demonstrated that MA administered to lactating mothers is detectable not only in the blood and brain of injected rat mothers but also in breast milk, which had been collected from the stomach of pups. Thus, MA diluted in maternal breast milk could potentially affect the postnatal development of lactating pups. Determining if there is a difference between direct and indirect MA administration on cognition in adult female rats was the next aim of the present study.

Sex hormones play a role in neural circuits and synaptic plasticity differences between males and females. It is well known that females are more sensitive to the effects of drug abuse than males, and that sensitivity to drugs increases with rising levels of estrogen during the female estrous cycle (Simpson et al., 2012). A study by Bisagno et al. (2003b) showed that females treated with MA had worse spatial abilities than males. A study by Warren and Juraska (1997) reported that hidden platform MWM performance was better in female rats during the proestrus phase than during diestrus. All these facts underline possible variations in the effect of MA on cognitive function in male and female rats as well as variations associated with the female rat estrous cycle.

To summarize, the present study aimed to investigate: (1) the effect of indirect and direct neonatal MA exposure; and (2) the effect of adult MA administration on cognitive abilities (spatial learning and memory) in adult female rats exposed to MA. To test cognitive functions, we used the hidden platform acquisition test in the MWM, one of the most widely used tasks in behavioral neuroscience for studying how substances/lesions affect allocentric navigation, a hippocampal-dependent form of learning and memory (Morris et al., 1982).

## Materials and Methods

The procedures used in this study were reviewed and approved by the Institutional Animal Care and Use Committee and meet the Czech Government Requirements under the Policy of Human Care of Laboratory Animals (No. 86/609/EEC) and with the subsequent regulations of the Ministry of Agriculture of the Czech Republic.

### Prenatal and Postnatal Animal Care

Adult female (250–300 g) Albino Wistar rats were purchased from Velez (Prague, Czech Republic, bred by Charles River Laboratories International, Inc.) and housed 4–5 per cage in a temperature-controlled (22–24°C) colony room using a standard 12 h light/dark cycle (lights on at 06:00). Before testing, animals were left undisturbed for 1 week with food and water *ad libitum*. After the acclimation period, the females were weighed and smeared, using vaginal lavage, to determine the phase of their estrous cycle. Females at the onset of the estrous phase of the estrous cycle were housed overnight with a sexually mature male (always one female and one male per cage) (Šlamberová et al., 2006). On the following day, the females were smeared for the presence of sperm and returned to their home cage. This day (the day after fertilization) was designated as Day 1 of gestation (GD 1). Dams were randomly assigned to MA-treated (MA) and saline-treated (S, isotonic saline) groups.

Pregnant females were weighed daily until delivery. On Day 21 of gestation (GD 21), females were removed from group cages and placed into maternity cages (one female/cage). Delivery occurred between days GD 22–23.

A total of 64 dams were used in the experiment. On PD 1, the number of pups in each litter was adjusted to 12. Whenever possible, the same number of male and female pups was kept in each litter. Between PD 1–11 pups received treatment as described below. On PD 21, the pups were weaned and housed in groups separated by sex. Light/dark cycle of the animals was reversed with lights-off at 06:00. Animals were left undisturbed until adulthood. In the present experiment, only females were tested (one female from each dam), while other females and males were used in other studies.

### Experimental Groups

Litters were divided into groups as shown in Table 1. One half of the offspring were treated indirectly *via* the breast milk of injected mothers, and the other half were treated directly by MA injection subcutaneously (s.c.). In the group with indirect exposure, mothers received MA (5 mg/ml/kg) or S (1 ml/kg) between PD 1–11. In the group with direct exposure none of the mothers were treated (i.e., no MA and no S). Instead, progeny were either: (1) treated with injected MA (5 mg/ml/kg); or (2) served as controls and received sham injections (no saline, just a needle stick) on PD 1–11. We used sham controls because our previous unpublished data showed that newborns injected with saline died at higher rates than MA injected pups.

**Table 1:** Assignment of the animals to individual groups according to the schedule and the type of neonatal (PD 1–11) indirect (i) and direct (d) exposure vs. acute treatment in adulthood.

Neonatal exposure				
Adult treatment	(i) Indirect		(d) Direct	
	iS (1 ml/kg)	iMA (5 mg/ml/kg)	dS (needle stick)	dMA (5 mg/ml/kg)
SA	iS/SA	iMA/SA	dS/SA	dMA/SA
(1 ml/kg)	*n* = 8	*n* = 8	*n* = 8	*n* = 8
MA	iS/MA	iMA/MA	dS/MA	dMA/MA
(1 mg/ml/kg)	*n* = 8	*n* = 8	*n* = 8	*n* = 8
Total number of animals				*n* = 64

*Total number of female rats used in experiment was 64; individual group accounted: 8 animals. Adult long-term treatment started on the day of beginning of Morris Water Maze Test and continues for subsequent 12 days. The MA dose of 5 mg/ml/kg was used during early lactation period and 1 mg/ml/kg was used for acute treatment in adulthood (Rambousek et al., 2014; Šlamberová et al., 2006; Hrebíčková et al., 2016)*.

The direct dose of MA for injection was chosen based on findings of MA levels seen in fetuses of drug-abusing women (Acuff-Smith et al., 1996; Šlamberová et al., 2005; Rambousek et al., 2014); additionally, MA exposure through maternal breast milk is similar in both rats and humans (Behnke and Smith, 2013; Rambousek et al., 2014). Physiological saline solution (0.9% NaCl) and d-Methamphetamine hydrochloride were purchased from Sigma–Aldrich (Czech Republic).

### Morris Water Maze

Adult female rats (PD 70–90) were used to test spatial learning and memory in the Morris Water Maze Test (MWM). Testing was performed under constant light conditions and a water temperature of 25°C. Based on their neonatal exposure, rats were divided into four groups: (1) iMA (indirect methamphetamine) group; (2) iS (indirect saline) group exposed indirectly *via* maternal breast milk; (3) dMA (direct methamphetamine); and (4) dS (direct sham) group exposed to sham needle sticks (Table 1). Thus, 32 indirectly exposed female rats (*n* = 16 with iS exposure + *n* = 16 with iMA exposure) and 32 directly exposed female rats (*n* = 16 with dS neonatal exposure + *n* = 16 with dMA neonatal exposure) were used in this study.

The MWM tests had three experimental phases: Place Navigation Test (Learning) on Days 1–6, Probe Test on Day 8, and Memory Recall Test (Memory) on Day 12 (Schutová et al., 2008). Each day, during the 12 days of the experiment, animals received injections of methamphetamine (MA; 1 mg/ml/kg) or saline (S; 1 ml/kg). For more details see Table 1. Animals were injected immediately after finishing a test on days involving swimming and at the same time on days without any swimming (Day 7 and Days 9–11). A low dose of MA was chosen because it does not lead to stereotypies, unlike the higher doses of 5 mg/ml/kg used during lactation (Šlamberová et al., 2006).

The Place Navigation Test (i.e., hidden platform acquisition test), performed on Days 1–6, was used to evaluate the ability of the animal to learn the specific location of a hidden platform. The concept behind the test is that the animal must learn to use environmental cues to navigate a direct path to a hidden platform when started from different random locations around the perimeter of the tank. The platform was placed in a fixed position, 1 cm under the water surface, making it invisible to a swimming rat. Four starting positions were designated around the rim of the maze: north (N), south (S), east (E), west (W), dividing the maze into four quadrants. The platform was always located in the N-E quadrant. Various pictures (environmental cues) were hung on the walls and could be used by rats as extra-maze cues. An animal was expected to find the hidden platform within 60 s. If the animal was not able to find the platform, it was manually guided to the platform, where it remained for 30 s. Each rat performed 8 trials per day starting from four different positions, with 30 s intervals (rest periods) between trials. Rat performance was tracked automatically using an EthoVision XT16 (Noldus Information Technology, The Netherlands) video-tracking system. After finishing all trials on an experimental day, animals were dried with a towel and injected with either MA (1 mg/ml/kg) or S (1 ml/kg) according to their group (Table 1). The animal was then returned to its home cage and remained undisturbed until the next experimental day.

The Probe Test was conducted on the 8th day of the experiment. Before the Probe Test trials, the platform was removed. The start position for all rats was the north (N) position, which was the nearest location to where the platform had been positioned (see “Place Navigation Test” section below). The animal was left to swim in the maze for 60 s. The quadrant preference and spatial strategy used during swimming in the probe trial assessed the ability to remember a spatial map.

The Memory Recall Test was performed on the 12th day of the experiment. This test determines if the animal can remember the position of the hidden platform, which was placed in the same position as in the learning phase (Place Navigation Test). Each rat performed 8 trials starting from four different positions (N, S, E, and W) and each trial lasted 60 s.

The visible platform was not included as a trial to compare learning skills and motivation, and therefore is a limitation of the study.

The following main parameters were evaluated with the use of the EthoVision program: the latency of platform acquisition [s], distance traveled (the length of the swim-path) [cm], search error (“cumulative distance” from platform throughout a trial) [cm], and the speed of swimming (“velocity”) [cm/s]. All parameters evaluated in the MWM test are summarized in Table 2.

**Table 2:** Parameters analyzed in the morris water maze (MWM) test.

The place navigation test	The probe test	The memory recall test
Latency of platform acquisition [s]	Distance traveled [cm]	Latency of platform acquisition [s]
Distance traveled (the length of the swim-path) [cm]	Number of platform crossing	Distance traveled [cm]
Search error (cumulative distance) [cm]	Duration of presence in the quadrant	Search error (cumulative distance) [cm]
Speed of swimming [cm/s]	where the platform was located [s]	Speed of swimming [cm/s]
	Speed of swimming [cm/s]	

### Analysis of Search Strategies

**Figure 1:**
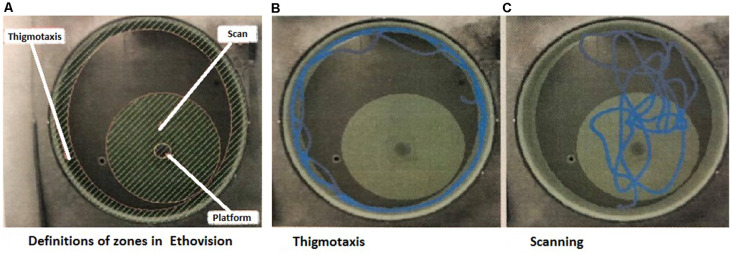
Snapshots from the program used for evaluation of strategies. **(A)** Definition of the arena with zones of thigmotaxis and scanning. In the middle of the Scanning zone is a hidden platform—the aim of the search. **(B)** Example track of an animal, which spent the majority of the time in thigmotaxis. **(C)** Example track of an animal, which spent the majority of time scanning (Macúchová et al., 2017).

A study by Janus (2004) demonstrated that swimming strategies are important signs of an animal’s ability to show spatial learning and are not just a random search for the platform. The evaluation of search strategies was modified from our previous protocol (Hrebíčková et al., 2014 and Macúchová et al., 2017), to allow digital analysis with the EthoVision XT16 system. In this study, we assessed two main search patterns (Macúchová et al., 2017; Figure 1): (1) thigmotaxis (wall-hugging)—a persistent swim along the wall of the pool, in an area of 30 cm from the wall; and (2) scanning—swimming over the central area of the pool (1 m in the diameter with the platform in the center). Swim paths for each rat during the Place Navigation Test (Days 1, 3, and 6) and Memory Recall Test (Day 12) were analyzed and both strategies were counted as the percentage of time using each strategy.

### Statistical Analyses

All data from the MWM Test were first assessed to determine the normality of distribution and homogeneity of variance and if data were parametric or non-parametric.

For statistical analyses of data, we used Statistica 12. Following statistical test were used in the three experimental phases of the MWM: (1) in the Place Navigation Test—three-way ANOVA (*Neonatal exposure × Treatment in adulthood × Application*) with multilevel repeated measure (*Days × Trials/day*); (2) in the Probe Test—three-way ANOVA (*Neonatal exposure × Treatment in adulthood × Application*); and (3) in the Memory Recall Test—three-way ANOVA (*Neonatal exposure × Treatment in adulthood × Application*) with repeated measure (*Trials*). The Bonferroni test was used for post-hoc comparisons.

### Estrous Cycle Determination

Since learning and memory in females can differ depending on the phase of the estrous cycle (Becker et al., 1982), the phase of the estrous cycle was determined for each female in the morning each day during the testing (Marcondes et al., 2002; Macúchová et al., 2017). To better show ovarian hormone-induced differences, only two contrast phases of the estrous cycle were used in the present study: metestrus/diestrus as a phase of low ovarian hormone levels and proestrus/estrus as a phase with high ovarian hormone levels (Simpson et al., 2012; Hrebíčková et al., 2017). The smear was examined using light microscopy. Diestrus or proestrus was determined based on vaginal smear cytology (i.e., diestrus has many leukocytes and very few cornified cells, while proestrus has some nucleated epithelial cells (Marcondes et al., 2002). The estrous cycle of a female rat lasts for 4–5 days.

The effect of the estrous cycle on cognition was a factor that we tried to include in the statistics but was without the effect, and for greater clarity in the graphs and the study itself, we decided not to include it as a result of the work. For more details see “Discussion” section.

## Results

### Main Effects of Neonatal Exposure and Acute Treatment in Adulthood

#### The Place Navigation Test

**Figure 2:**
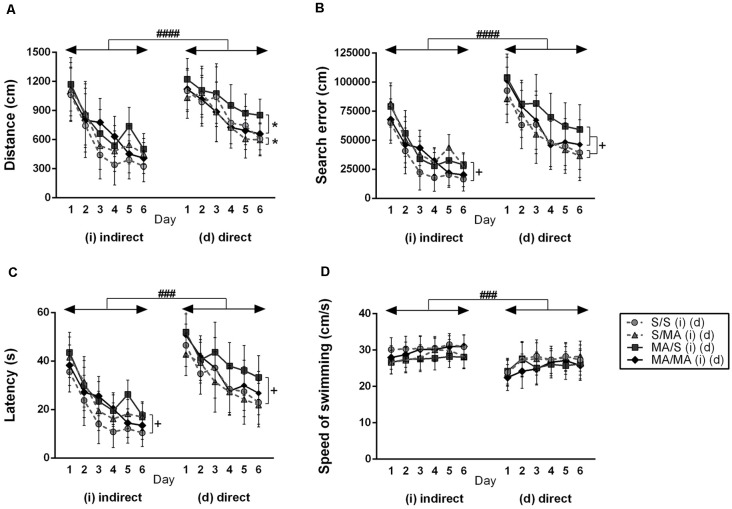
Effect of neonatal (indirect/direct) and acute methamphetamine (MA) application on performance of adult females on individual days in the Place Navigation Test. Results are presented as mean ± SEM, *n* = 8. **(A)** Distance traveled: direct exposure *p < 0.05 — acute MA treatment in adulthood: MA groups swam shorter distances than S-treated females; ^####^*p* < 0.0001 pups exposed directly to MA by injections swam longer distances than animals with indirect MA exposure, **(B)** search error: ^+^*p* < 0.05 neonatal indirect and also direct MA exposure leads to increased search errors; ^####^*p* < 0.0001 pups exposed directly to MA by injections did more search errors than animals with indirect MA exposure, **(C)** latency: ^+^*p* < 0.05 neonatal indirect and also direct MA exposure leads to increased time to find the hidden platform; ^###^*p* < 0.001 pups exposed directly to MA by injections need more time to reach the hidden platform than animals with indirect MA exposure, **(D)** speed of swimming.

In order to see if indirect and direct neonatal MA exposure affects learning ability, all tested animals were analyzed (S vs. MA groups). The results did not reveal any main effect of indirect neonatal MA exposure on distance traveled (Figure 2A; *F*_(1,28)_ = 2.97; *p* = 0.10), search errors (Figure 2B; *F*_(1,28)_ = 2.07; *p* = 0.16), and speed of swimming (Figure 2D; *F*_(1,28)_ = 0.72; *p* = 0.40). Only latency was increased after neonatal iMA exposure (Figure 2C; *F*_(1,28)_ = 4.50; *p* < 0.05). Directly MA-exposed females did not differ in distance traveled (Figure 2A; *F*_(1,28)_ = 2.43; *p* = 0.13), latency (Figure 2C; *F*_(1,28)_ = 5.98; *p* = 0.20), and velocity (Figure 2D; *F*_(1,28)_ = 1.80; *p* = 0.19). Direct MA exposure increased search errors (Figure 2B; *F*_(1,28)_ = 5.10; *p* < 0.05).

No effect of acute MA treatment has been seen in the distance traveled (Figure 2A; *F*_(1,28)_ = 0.28; *p* = 0.60), search error (Figure 2B; *F*_(1,28)_ = 0.62; *p* = 0.44), latency (Figure 2C; *F*_(1,28)_ = 0.49; *p* = 0.49), and velocity (Figure 2D; *F*_(1,28)_ = 0.02; *p* = 0.97) in the animals exposed neonatally through breast milk. The main effect of acute MA treatment was found on the length of the swim-path of dMA females. MA application in adulthood decreased distance traveled compared to saline-treated controls (Figure 2A; *F*_(1,28)_ = 4.22; *p* < 0.05). Animals exposed directly did not differ in the search error (Figure 2B; *F*_(1,28)_ = 1.43; *p* = 0.24), latency (Figure 2C; *F*_(1,28)_ = 1.73; *p* = 0.12), and velocity of swimming (Figure 2D; *F*_(1,28)_ = 0.09; *p* = 0.76).

Detailed analysis of strategies using during Place Navigation Test showed that indirect MA exposure had no effect on thigmotaxy (Figure 4A; *F*_(1,28)_ = 2.30; *p* = 0.14) and also on scanning (Figure 4B; *F*_(1,28)_ = 1.17; *p* = 0.74). Direct MA exposure did not affect any strategy used by females in learning phase of MWM test: thigmotaxy (Figure 4A; *F*_(1,28)_ = 2.19; *p* = 0.62) and scanning (Figure 4B; *F*_(1,28)_ = 5.17; *p* = 0.09).

When we compared the main effects of indirect vs. direct neonatal MA on learning abilities of adult females, statistical analyses showed that dMA exposure had a more significant impact on learning than iMA. dMA females swam longer distances (Figure 2A; *F*_(5,280)_ = 30.85; *p* < 0.0001), had many more search errors (Figure 2B; *F*_(5,280)_ = 52.78; *p* < 0.0001), needed more time to find the hidden platform (Figure 2C; *F*_(5,280)_ = 59.47; *p* < 0.001), and swam much slower (Figure 2D; *F*_(5,280)_ = 14.56; *p* < 0.001) than iMA females. Detailed analysis of strategies of swimming confirms these results. dMA females spent more time in thigmotaxis (Figure 4A; *F*_(9,168)_ = 21.74; *p* < 0.01) on Day 3 and 6 compared to iMA females.

Table 3 summarizes the main effect of neonatal MA exposure (iM/dM) on measurements of the Place Navigation Test.

**Table 3:** Effect of neonatal indirect and direct methamphetamine exposure on the performance of females in the place navigation test and memory recall test.

Neonatal MA		Place navigation test				Memory recall test			
		Distance	Search error	Latency	Velocity	Distance	Search error	Latency	Velocity
	indirect	–	↑	↑	–	–	–	–	–
		–	↑	↑	–	↑	–	↑↑	↓

*– no effect; ↑ *p* < 0.05; ↑↑ *p* < 0.01; ↓ *p* < 0.05*.

#### The Probe Test

Neonatal indirect MA exposure had no effect on distance traveled (*F*_(1,28)_ = 0.03; *p* = 0.86) and speed of swimming (*F*_(1,28)_ = 0.21; *p* = 0.65). Also direct MA exposure injected to pups did not affect distance (*F*_(1,28)_ = 0.01; *p* = 0.93) and velocity (*F*_(1,28)_ = 0.01; *p* = 0.98) during Probe Test.

MA treatment in adulthood increased only speed of swimming (*F*_(1,28)_ = 4.28; *p* < 0.05) of females with indirect exposure. Distance traveled in these group of animals has not been changed (*F*_(1,28)_ = 3.12; *p* = 0.09). Acute dose of MA did not affect distance traveled (*F*_(1,28)_ = 0.74; *p* = 0.40) and velocity (*F*_(1,28)_ = 1.88; *p* = 0.18) in females exposed neonatally by direct injection.

#### The Memory Recall Test

**Figure 3:**
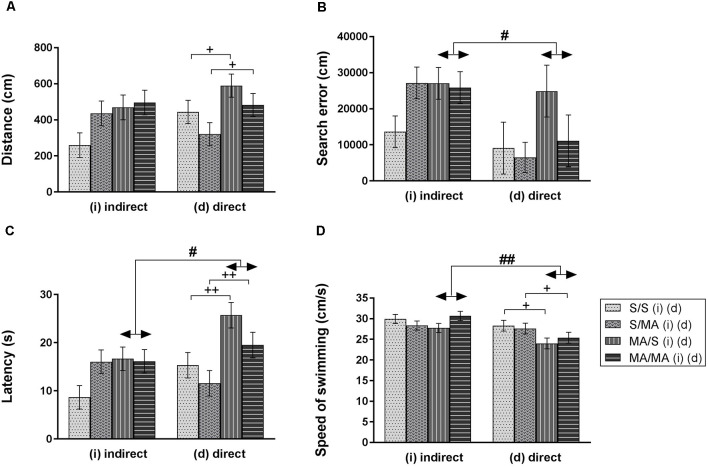
Effect of neonatal (indirect/direct) and acute MA application on performance of adult females in the Memory Recall Test. Results are presented as mean ± SEM, *n* = 8. **(A)** Distance traveled: neonatal direct MA (dMA) increased distance traveled ^+^*p* < 0.05, **(B)** search error: ^#^*p* < 0.05 pups exposed directly to MA by injections did fewer search errors than animals with indirect MA exposure, **(C)** latency: ^++^*p* < 0.01 neonatal direct MA exposure leads to increased time to find the hidden platform; ^#^*p* < 0.05 pups exposed directly to MA by injections need more time to reach the hidden platform than animals with indirect MA exposure, **(D)** speed of swimming: ^+^*p* < 0.05 neonatal direct MA exposure leads to decreased speed of swimming; ^##^*p* < 0.01 pups exposed directly to MA by injections swam slower than animals with indirect MA exposure.

Statistical analyses did not reveal any main effect of iMA on measurements of Memory test of adult females (Figure 3): distance traveled (A) (*F*_(1,28)_ = 3.88; *p* = 0.06), search error (B) (*F*_(1,28)_ = 1.91; *p* = 0.18), latency (C) (*F*_(1,28)_ = 2.75; *p* = 0.11), and velocity (D) (*F*_(1,28)_ = 0.04; *p* = 0.95). The Memory Recall Test only revealed differences in performance of neonatal dMA adult female rats. Figure 3 shows main effect of dMA exposure on tested measurements. Direct neonatal MA exposure leads to increased length of swim trajectories (A) (*F*_(1,28)_ = 5.74; *p* < 0.05), latency to reach the hidden platform (C) (*F*_(1,28)_ = 11.94; *p* < 0.01), and decreased swimming speed (D) (*F*_(1,28)_ = 6.16; *p* < 0.05). The measurement search error has not been affected by dMA (Figure 3B; *F*_(1,28)_ = 2.02; *p* = 0.17).

Acute MA treatment did not affect the performance of adult females on the 12th day of the MWM Test (Figure 3). The statistical results for group of indirect exposure: distance traveled (A) (*F*_(1,28)_ = 2.16; *p* = 0.15), search error (B) (*F*_(1,28)_ = 1.99; *p* = 0.17), latency (C) (*F*_(1,28)_ = 1.98; *p* = 0.17), and speed of swimming (D) (*F*_(1,28)_ = 0.34; *p* = 0.56). Group of direct neonatal exposure did not reveal any significant difference in the distance traveled (A) (*F*_(1,28)_ = 3.26; *p* = 0.08), search error (B) (*F*_(1,28)_ = 1.30; *p* = 0.26), latency (C) (*F*_(1,28)_ = 3.52; *p* = 0.07), and velocity (D) (*F*_(1,28)_ = 0.07; *p* = 0.79).

**Figure 4:**
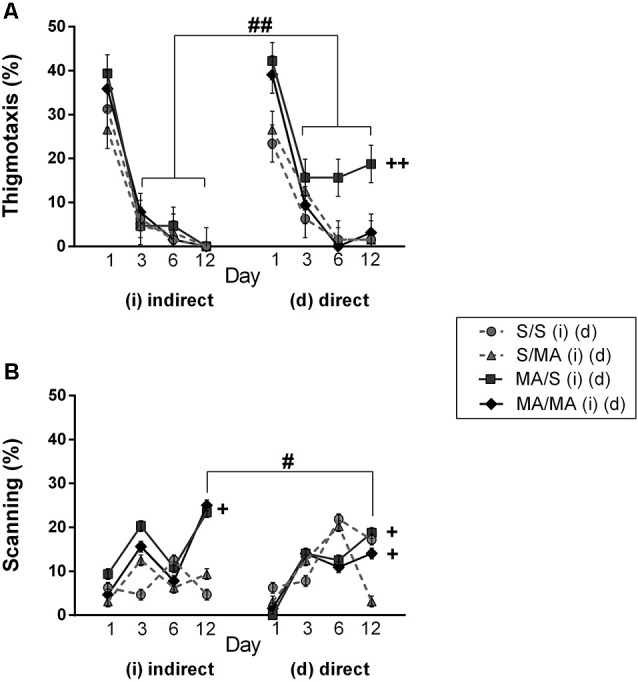
Effect of neonatal (indirect/direct) and acute MA application on the percentage of time spent in search strategies on individual days of the learning phase (Day 1, 3, 6) and also Day 12—Memory Recall Test of adult females. **(A)** Thigmotaxis: ^++^*p* < 0.01 neonatal direct MA exposure leads to higher thigmotaxis on Days 3, 6, and 12; ^##^*p* < 0.01 pups exposed directly to MA by injections displayed more thigmotaxis than animals with indirect MA exposure. **(B)** Scanning: ^+^*p* < 0.05 neonatal direct and indirect MA exposure lead to decreased use of scanning relative to control groups; ^++^*p* < 0.01 neonatal direct and indirect MA exposure leads to lower scanning on the Day 12; ^#^*p* < 0.05 pups exposed directly to MA by injections displayed lower scanning than animals with indirect MA exposure.

Detailed analysis of search strategies on the 12th day of MWM test showed no effect of indirect neonatal MA exposure on percentage of use thigmotaxy (Figure 4A; *F*_(1,28)_ = 5.08; *p* = 0.09). Increased use of the scanning strategy (Figure 4B; *F*_(1,28)_ = 2.99; *p* < 0.05) was found after neonatal iMA exposure compared to the iS group. Moreover neonatal dMA exposure led to increased use of thigmotaxis (Figure 4A; *F*_(1,28)_ = 7.24; *p* < 0.01) and increased use of scanning (Figure 4B; *F*_(1,28)_ = 4.50; *p* < 0.05) relative to dS-treated females.

One of the aims of this study was to determine if there was a difference between indirect vs. direct neonatal MA exposure on memory of adult female rats. Statistical analyses showed main significant differences in patterns during the Memory Recall Test. Females exposed to neonatal dMA displayed fewer search errors (Figure 3B; *F*_(1,28)_ = 6, 50; *p* < 0.05), spent more time finding the hidden platform (Figure 3C; *F*_(1,28)_ = 7, 02; *p* < 0.05), and swam slower (Figure 3D; *F*_(1,28)_ = 8, 64; *p* < 0.01) than females exposed to iMA. Statistical analysis did not reveal any difference in the length of the swim-path (Figure 3A; *F*_(1,28)_ = 7.20; *p* = 0.09). Analyzes of strategies revealed that females after dMA exposure spent a higher percentage of time in thigmotaxis (Figure 4A; *F*_(1,28)_ = 20.14; *p* < 0.01) and a lower percentage of time scanning (Figure 4B; *F*_(1,28)_ = 2.50 *p* < 0.05) than females exposed to iMA.

Table 3 summarizes the main effect of neonatal MA exposure (iM/dM) on measurements of the Memory Recall Test.

### Interaction Effect of Neonatal Exposure and Acute Treatment in Adulthood

#### The Place Navigation Test

Statistical analyses of individual days of learning phase showed interaction effect of neonatal exposure and acute treatment in adulthood. Indirect neonatal MA exposure leads to increased search errors (Figure 2B; *F*_(5,280)_ = 7.08; *p* < 0.05) and latency (Figure 2C; *F*_(5,280)_ = 6.34; *p* < 0.05) in females treated in adulthood with saline. Distance traveled (Figure 2A; *F*_(5,280)_ = 1.52; *p* = 0.19) and velocity (Figure 2D; *F*_(5,280)_ = 0.36; *p* = 0.87) have not been changed after neonatal indirect exposure and acute treatment in adulthood. No differences appeared in distance traveled (Figure 2A; *F*_(5,280)_ = 0.38; *p* = 0.86), search error (Figure 2B; *F*_(5,280)_ = 0.73; *p* = 0.60), and velocity (Figure 2D; *F*_(5,280)_ = 0.97; *p* = 0.44) in females after direct exposure and adult treatment. Only latency to reach the hidden platform (Figure 2C; *F*_(5,280)_ = 5.99; *p* < 0.05) was longer in females after direct neonatal MA exposure and acute S treatment in adulthood.

Analyzing search strategies on individual days of the learning phase showed no differences in interaction effect of neonatal indirect exposure and acute treatment in adulthood in percentage of use thigmotaxy (Figure 4A; *F*_(9,168)_ = 1.55; *p* = 0.15) and strategy (Figure 4B; *F*_(9,168)_ = 1.28; *p* = 0.32). Interaction effect was found in females exposed to dMA during neonatal period and treated with S in adulthood (dMA/S). This group of females displayed higher thigmotaxis (Figure 4A; *F*_(9,168)_ = 8.45; *p* < 0.01) on the Day 3 and 6 compared to the control group (S/S). No interaction effect was found in percentage use of scanning (Figure 4B; *F*_(9,168)_ = 4.15; *p* < 0.08).

#### The Probe Test

Neonatal indirect exposure vs. acute application in adulthood did not affect distance traveled (*F*_(1,28)_ = 1.79; *p* = 0.08) and velocity (*F*_(1,28)_ = 1.27; *p* = 0.27). Statistical analyses showed interaction effect of neonatal direct MA exposure and acute MA treatment in adulthood. dMA females with an acute MA exposure (dMA/MA) swam longer trajectories to find the hidden platform than dMA/S animals (*F*_(1,28)_ = 10.75; *p* < 0.01). Speed of swimming did not changed in females after neonatal direct exposure vs. acute treatment in adulthood (*F*_(1,28)_ = 1.04; *p* = 0.32).

#### The Memory Recall Test

Statistical analyses of interaction effect of neonatal exposure and adult treatment in individual trials showed no difference of any of tested measurements. In the group of females exposed indirectly the distance traveled (Figure 3A; *F*_(7,196)_ = 1.70; *p* = 0.11), search error (Figure 3B; *F*_(7,196)_ = 1.22; *p* = 0.29), latency (Figure 3C; *F*_(7,196)_ = 1.55; *p* = 0.15), and velocity (Figure 3D; *F*_(7,196)_ = 1.42; *p* = 0.20) have not been changed. We found the same results in the group of direct neonatal exposure in which distance traveled (Figure 3A; *F*_(7,196)_ = 1.07; *p* = 0.38), search error (Figure 3B; *F*_(7,196)_ = 0.93; *p* = 0.49), latency (Figure 3C; *F*_(7,196)_ = 1.23; *p* = 0.29), and velocity (Figure 3D; *F*_(7,196)_ = 1.16; *p* = 0.32) not been affected, too.

Analyzing search strategies on 12th day of MWM test did not revealed significant differences in the interaction effect of neonatal and acute treatment. In the group of neonatal indirect exposure the percentage of use thigmotaxy (Figure 4A; *F*_(7,196)_ = 4.19; *p* = 0.09) and scanning (Figure 4B; *F*_(7,196)_ = 2.84; *p* = 0.31) did not affected. Also in the group exposed directly both strategies used find to hidden platform not been affected, thigmotaxy (Figure 4A; *F*_(7,196)_ = 2.43; *p* = 0.014) and scanning (Figure 4B; *F*_(7,196)_ = 3.15; *p* = 0.09).

## Discussion

The present study examined cognitive functions of adult female rats neonatally exposed to the same drug (5 mg/ml/kg) in two different ways (indirectly/directly) during PD 1–11.

The first goal of the present study was to investigate the potential effects of neonatal indirect MA exposure *via* breast milk and direct MA exposure by injection during PD 1–11 on spatial learning and memory formation of adult female rats. Our results showed that both types of early neonatal MA exposure worsened the performance of females during water maze testing. Females exposed to neonatal MA displayed more search errors and needed more time to find the hidden platform during navigation tasks. Learning alterations, which manifested as a disturbance in the consolidation process involving the “trajectory to the hidden platform,” were shown by poorer results on the Probe and Memory Recall tests. Because previous studies showed that two animals may have similar escape latencies or length of trajectories during the trials, while having markedly different performances (Gallagher et al., 1993; Janus, 2004), we also analyzed the search strategies used to find the hidden platform. Analysis of search strategies showed impaired cognition, which supports our other findings. Specifically, females with direct neonatal MA exposure spent more time using thigmotaxis and scanning and indirect neonatal MA exposure spent more time using scanning instead of using a direct trajectory to the hidden platform during advanced phases of MWM (Janus, 2004).

Moreover, we found that females exposed to neonatal MA swam slower during memory tests than control females. Speed of swimming can be used as a measure of motivation to find the hidden platform (Lubbers et al., 2007). Motivation is assumed to be mediated by the meso-accumbens dopaminergic system (Salamone and Correa, 2002), which starts to develop, as does the hippocampus, between GD 12 and PD 19–20 (Bayer et al., 1993; Rice and Barone, 2000; Jablonski et al., 2016). Results from the present study suggest that extensive deficits in learning and memory may be linked to the negative impact of MA exposure on the development of the meso-accumbens and on hippocampal dopamine production. Our results correspond with our previous studies, in which we demonstrated increased deficits in the learning ability of male rats after postnatal MA exposure during PD 1–11 (Hrebíčková et al., 2016) and PD 1–21 (Hrubá et al., 2010). There are other studies describing the impairments of MA treatment during sensitive periods, e.g., PD 6–15 and PD 11–20, in which MA was applied in doses of 10–25 mg/kg appeared more sensitive to MA administration, whereas PD 1–10 or PD 21–30 were less or not sensitive to MA administration (Williams et al., 2003b; Schaefer et al., 2008; Vorhees et al., 2009; Jablonski et al., 2016). Vorhees et al. (1994a) showed that MA administration during PD 1–10 at a dose of 30 mg/kg/day induced changes only in the locomotor activity of adult rats tested in a water maze, while administration during PD 11–20 at a dose of 40 mg/kg divided into 4 doses/day reduced memory performance on probe trials (Vorhees et al., 1994a,b). Another study showed that neonatal administration of MA at doses of 5, 10, or 15 mg/kg 4 × day during PD 11–20 produced spatial learning and memory impairments (Williams et al., 2003a). From our results and the results of others, it seems that early postnatal MA exposure worsens cognition in rats.

As in our other recent studies (Hrebíčková et al., 2016, 2017; Ševčíková et al., 2017), the present study was interested in determining which route of neonatal MA exposure (indirect or direct) has the most significant impact on behavior/learning/memory in adult rats. Statistical analyses showed that direct MA exposure impeded learning processes and memory formation to a greater extent than did indirect MA. Moreover, females with direct MA exposure had a higher incidence of thigmotaxis across the days of learning than females with indirect MA. Our previous experiments (MWM, Social Interaction Test; Hrebíčková et al., 2016, 2017) demonstrated a larger effect of direct vs. indirect MA administration, which were similar to our present results. The reason why can only be hypothesized. One of our assumptions is that direct neonatal MA injection has an almost instantaneous effect, while ingestion of MA transported *via* breast milk is absorbed slowly into the body of pups. In indirect MA exposure, the drug is metabolized in the body of the mother. The half-life of MA in rats is 70 min (Cho et al., 2001; Melega et al., 2007). MA-treated mothers display more activities of self-care and pay less attention to their pups immediately after drug exposure, which we showed in a study by Ševčíková et al. (2017). Suckling pups do not have the chance to suck until the effect of the drug on the mother has diminished, therefore, they are exposed to MA at lower doses than pups with direct MA injections; we know that maternal care is essential for normal somatic growth and neurodevelopment of the pups. There was a study that showed that differences in maternal care during the first week of postnatal life could influence hippocampal development and function (Liu et al., 2000). To work out the details of our hypothesis, future experiments that examine MA concentrations in the brains and blood of pups, as well as in breast milk after direct and indirect MA exposure are needed.

The second goal of the present study was to determine the effect of acute MA (1 mg/ml/kg) treatment on cognition in adult female rats. Administration of MA did not reveal any significant impairment in performance on the MWM Test. There are studies, which are consistent with the presented results, demonstrating that chronic MA treatment in adulthood does not induce changes in cognitive functions (Simões et al., 2007; Belcher et al., 2008). Some preclinical studies have reported that acute MA administration at low doses (0.1–0.4 mg/ml/kg) produced improvements in cognitive processing (Grilly and Loveland, 2001), while higher doses of MA (3 mg/ml/kg and more) induced impairment in spatial learning and memory (Robbins, 2002). As mentioned by Meredith et al. (2005) in their review, the severity of neurocognitive deficits is dose- and frequency-dependent.

Even though the present study tested only female offspring, our previous study examining adult males after the same early postnatal exposure (PD 1–11) allows us to discuss possible sex differences. It is well known that there are significant sex differences in the molecular mechanisms of learning and memory at every level of intracellular signaling, which includes receptors, second messengers, and even histone modification (Keiser and Tronson, 2016). It can be seen, e.g., in strategies used by males and strategies used by females on the spatial acquisition task; females used thigmotaxis more often compared to males (Jonasson, 2005). Moreover, other studies have confirmed that estrogen and progesterone can stimulate dopamine function, which resulted in a modulation of hippocampal dendritic spine density and long-term potentiation during proestrus and estrus compared to diestrus (Becker et al., 1982, 2001; Woolley and McEwen, 1992; Warren and Juraska, 1997; Becker and Hu, 2008). A study by Bisagno et al. (2003a) showed that females treated with MA had worse spatial abilities than males. A study by Warren and Juraska (1997) reported that hidden-platform MWM performance was better during proestrus than during diestrus. Due to these facts, the minor aim of our study was to try to determine the potential effect of the estrous cycle on performance in MWM after drug treatment. The effect of the estrous cycle on cognition was a factor that we tried to include in the statistics but was without the effect, and for greater clarity in the graphs and the study itself, we decided not to include it as a result of the work. Our previous study of Macúchová et al. (2017) confirms our findings of no effect of the estrous cycle on cognition of adult females. This issue will be studied in more detail in one of our upcoming studies, which will test the effects of prenatal MA exposure during gestation and acute MA treatment on cognition with respect to the estrous cycle in more details.

## Conclusion

The analyses presented in this study are unique in that they compare the effects of indirect and direct neonatal MA exposure on cognitive functions in adult female rats. Our results show that both types of neonatal MA exposure impair cognitive functions and that direct MA exposure has a more negative impact on spatial learning and memory than indirect exposure.

## Data Availability Statement

The original contributions presented in the study are included in the article, further inquiries can be directed to the corresponding author.

## Ethics Statement

The animal study was reviewed and approved by Institutional Animal Care and Use Committee.

## Author Contributions

IP-H carried out the experiments, drafted statistic analysis, and wrote the manuscript. MS participated in the experiments. RŠ conceived of the study, coordinated the study, and helped to draft the manuscript. All authors read and approved the final manuscript.

## Conflict of Interest

The authors declare that the research was conducted in the absence of any commercial or financial relationships that could be construed as a potential conflict of interest.
